# Application of the Acoustic Emission Method and Kolmogorov-Sinai Metric Entropy in Determining the Yield Point in Aluminium Alloy

**DOI:** 10.3390/ma13061386

**Published:** 2020-03-19

**Authors:** Katarzyna Panasiuk, Leslaw Kyziol, Krzysztof Dudzik, Grzegorz Hajdukiewicz

**Affiliations:** Faculty of Marine Engineering, Gdynia Maritime University, 81-225 Gdynia, Poland; l.kyziol@wm.umg.edu.pl (L.K.); k.dudzik@wm.umg.edu.pl (K.D.); g.hajdukiewicz@wm.umg.edu.pl (G.H.)

**Keywords:** acoustic emission, yield point, Kolmogorov-Sinai metric entropy, tensile test

## Abstract

This study analyzes the possibility of applying the acoustic emission method (AE) and the Kolmogorov-Sinai (K-S) metric entropy phenomenon in determining the structural changes that take place within the EN AW 7020 aluminum alloy. The experimental part comprised of a static tensile test carried out on aluminum alloy samples, and the simultaneous recording of the acoustic signal generated inside the material. This signal was further processed and diagrams of the effective electrical signal value (RMS) as a function of time were drawn up. The diagrams obtained were applied on tensile curves. A record of measurements carried out was used to analyze the properties of the material, applying a method based on Kolmogorov-Sinai (K-S) metric entropy. For this purpose, a diagram of metric entropy as a function of time was developed for each sample and applied on the corresponding course of stretching. The results of studies applying the AE and the K-S metric entropy method show that K-S metric entropy can be used as a method to determine the yield point of the material where there are no pronounced yield points.

## 1. Introduction

Like the majority of materials, metals emit elastic (acoustic) waves when under a load. These waves are emitted due to a large number of auxiliary phenomena, which is why they can be tested applying the Acoustic Emission (AE) method [1]. These phenomena include plastic deformations, i.e., dislocation slide or crystal twinning; the formation and growth of cracks and the destructive process; phase transitions [2,3]. One of the main sources of AE in metals is dislocation, particularly any movement that causes significant accelerations or delays. When the material is exposed to very high external loads, dislocation speed approximates the speed of sound. When the loads are smaller, the speed is much lower, i.e., from 1 to 50% of the speed of sound. Accelerations and delays of dislocation are very large and take place in very short time intervals: 10**^−^**^11^**–**10**^−^**^10^ s and in very small sections of 1**–**50 of crystalline network parameters [4,5,6,7]. Due to the dependence of AE on the degree of plastic deformation, the speed of its growth and the metal volume, in which this deformation develops, even with single-axial elongation, there are several typical varieties of acoustic emission characteristics as a function of mean effective electrical signal value (RMS). AE signal in the form of elastic waves is converted into electrical signal using piezoelectric converters [8,9].

By definition, acoustic emission is a transient elastic wave produced through the rapid release of energy accumulated in the material, and propagating micro-damages (growth of micro-apertures, movement of dislocation groups) in the material or caused by a process (friction, leaks, etc.) [10,11,12,13]. The AE method can be used to conduct continuous testing, during the operation of the tested objects. AE instruments record the signal which is generated in a given object during its normal operation or in the course of functional tests [14,15,16]. Each tested material with a homogeneous structure has its own resonance frequencies. By introducing impulse mechanical stimulation (loading this material) and examining the frequency structure of the acoustic emission signal, it is possible to detect microcracks by observing the change in the resonant frequencies of the object [17]. Acoustic waves are propagated in all directions from the source, so they can be recorded by one or more sensors installed on the object or element [18,19]. During propagation, AE waves are muffled, which reduces the distance, at which they can be detected. This distance depends on many factors, among others on the properties of the material, the geometry of the object and the level of acoustic background interference [8].

Acoustic emission (AE) is a widely-used method, applied in the monitoring of buildings and structures, as well as in testing materials [20]. This is exemplified by tests carried out on smooth samples and ones with a notch, as delivered and as in operation [21]. The study [22] proposes criteria for assessing the technical condition of bunker tanks, basing on AE signals. Whereas, studies in [23] present the application of the EA method in studying wood cracking across the fibers.

The paper proposes a new way of developing experimental data. It is an attempt to holistically look at the mechanism of error dynamics and measurement disturbances. The process of creating scattering of measurement data is undoubtedly very complex and it is difficult to sublime one parameter that would control this process. To overcome this difficulty, it is proposed to analyze data where measurement disturbances are treated as a non-linear dynamical system whose metric entropy is the key magnitude. The application of the Kolmogorov-Sinai (K-S) metric entropy in describing the mechanical properties of materials is based on a dynamic notion, whereby K-S entropy is the “entropy of a time unit” and is therefore non-negative, but can both increase and decrease [23,24,25,26,27]. The relationship between statistical mechanics and the chaos theory reflects the notion of K-S metric entropy, which was introduced by Kolmogorov in 1958 [28,29]. The essence of K-S metric entropy is that its nature is dynamic as it describes system movement typical for chaotic processes [30,31,32]. Metric entropy is a value that measures the instability of the dynamics of a system, i.e., expresses a method for the numerical description of chaos. It is based on the assumption that the qualitative changes which take place at the structural threshold separating the elastic state from the plastic state correspond to a specific measurement point. The energy dissipation which takes place in the system and the deterministic chaos of data related to this phenomenon causes the variability of entropy [25].

According to the second law of thermodynamics, the total entropy of an isolated system can never decrease over time, and will thus always approach positive values. A statistical interpretation of the second law of thermodynamics was coined by L. Boltzmann. He demonstrated that macroscopic state entropy S is proportional to the thermodynamic probability of this state W. This interrelation was expressed by Equation (1) [25]:

(1) S=k×lnW

where: k - Boltzmann's constant.

This means that the dynamics of a system leads to the formation of increasingly probable states as it approaches the maximum value of thermodynamic probability.

Kolmogorov relied on the concept of statistical information proposed by Shannon to describe the transmission of telecommunications signals. According to Shannon, the news that something should happen has no information. However, for independent events, information about them should be the sum of individual information. These conditions are met when the measure of information is a logarithmic function of probability. The essence of metric entropy is that it is dynamic because it describes the motion of the system, characteristic of chaotic processes.

In this method, it is essential to prepare the input data used to calculate metric entropy correctly. During the static tensile test, the tester must record the stress and the strain, and a column of quotients must be created at the same time. The minimum value of metric entropy in the vicinity of the passage from elastic to plastic state is marked by the point that separates individual states of the process [26]. 

Considering the subjective character of the current methods for determining the yield point, a procedure was proposed, consisting in determining the metric entropy of measurement data, treating the plasticity limit as a “critical point” that separates the elastic state from the plastic state in the process of deforming a structural material [33].

The notion of “critical point” used in this study was referred to specific points recorded during the tensile test, without the intention of applying the broader meaning of this notion, defined strictly in other physical theories. In this method, a decrease in the value of metric entropy is expected to occur at the plasticity limit, as caused by non-linearities related to a change in the structure of data in the vicinity of this passage from the elastic state to the plastic state, magnified by fluctuations related to the occurrence of deterministic chaos.

A recorded decrease in entropy is closely related to the dissipation of energy in the tested sample, and therefore to the noticeable change of dynamics of data recorded by the universal testing machine. A change in the dynamics of data is the material’s response to the load it is subjected to, and the structural changes which take place inside it. 

Based on the data obtained from the static tensile test of the AW-7020 alloy, sets of absolute elongation values (expressed in mm) for individual measuring points were created. These sets, for each of the samples are deformation courses as a function of time *ε (t)*. All tensile tests of the samples were carried out at a constant force speed F (force-controlled tests). The sets of absolute elongation values were used as input files to calculate metric entropy (entropy of measurement data). Entropy calculation by the K-S method consists in dividing the set of absolute elongation values for individual measuring points into number ranges. Next, the compartments are divided into sub-compartments and the entropy value of each compartment is calculated according to the principle defined by Kolmogorow-Sinai [28,29,33]:

(2) $$S=-\sum_{i=1}^{N}p_{i}\times \ln p_{i}$$

where: N - is the number of partitions into which a set of all possible results was divided; *pi* - is the probability of results occurrence in i-th partition, (while in the definition if *pln p* = 0 , if *p* = 0).

The yield point is one of the basic features of construction materials that is used in design engineering calculations. If the material had ideal elastic–plastic stretching characteristics, then determining the yield point would be obvious and unambiguous. However, many engineering plastics do not exhibit a stop in plasticity, and the elastic characteristics are not always linear. In this situation, additional assumptions and specific procedures were introduced to determine the yield point of the material.

Tests using the acoustic emission method as well as K-S metric entropy show that it is possible to determine the exact yield strength of materials without specifying their conventional values. In this topic, the metric entropy method K-S is unknown, and can be used to determine solid materials, not just metals and their alloys. Earlier studies have indicated that this point reflects the passage of the material from the elastic state to the plastic state and determines the yield point [25,32,33,34,35].

## 2. Materials and Methods

Rolled sheet metal, g = 12 mm thick, made of EN AW 7020 alloy, the chemical composition of which is presented in Table 1 was the tested specimen. The tensile testing specimen were cut across the direction of rolling.

**Figure 1:**
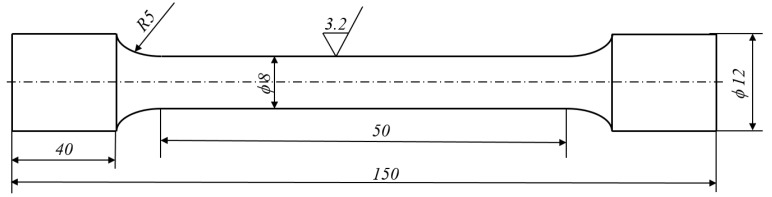
Shape and dimensions of samples for static tensile testing.

Samples were turned and prepared accordance with the norm. The shape and dimensions of the sample are presented in Figure 1, in accordance with PN-EN ISO 6892-1: 2016-09 [36]. 

The static tensile test was carried out on EN AW 7020 alloy samples placed in a universal hydraulic strength testing machine, type MPMD P10B, on TestXpert II software, version 3.61. by Zwick&Roell (Ulm, Germany), with a power range to 100 kN. The test was carried out on three samples.

**Figure 2:**
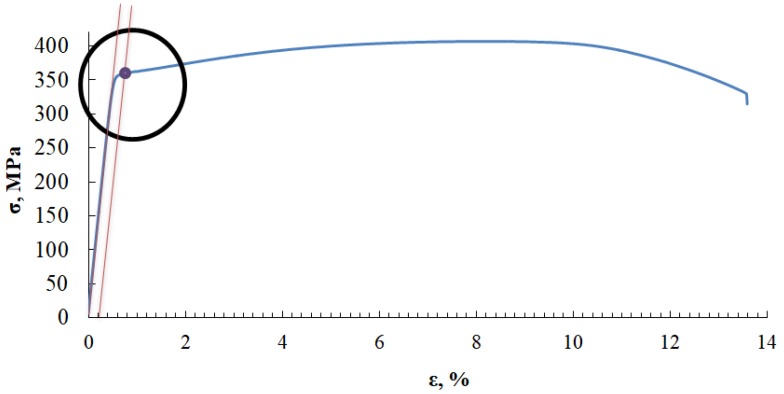
Diagram σ - ε for the analyzed Sample No. 3 made of AW 7020 alloy, with a marked fragment subject to further analysis, as well as with a point which corresponds to the arbitrary yield point R_0.2_.

**Figure 3:**
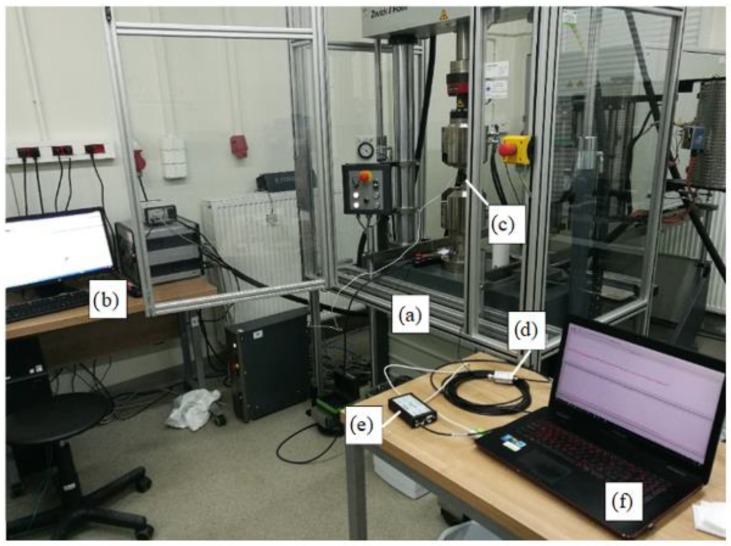
General view of the laboratory stand: (**a**) tensile stress machine, (**b**) tensile stress machine computer, (**c**) AE sensor, (**d**) preamplifier, (**e**) AE recorder, (**f**) AE computer.

For instance, Figure 2 presents the static characteristics of the tensile test carried out on Sample No. 3 made of EN AW 7020 alloy. The part of the diagram marked with a circle means that this part only will be studied. Material strength tests were carried out to study the characteristics of EA signals, and acoustic signals were simultaneously recorded. In the course of the static tensile test, individual examinations were conducted in a testing station comprising AE measurement instruments and a universal testing machine. An acoustoelectric sensor was installed on the tested sample to record the acoustic waves generated inside the material. These waves were converted by the sensor into electric signal and then recorded in the digital form by the recorder. The recorded signal was further processed and diagrams of the effective electrical signal value (RMS) as a function of time were drawn up. Figure 3 presents a photograph of general view of the laboratory stand.

**Figure 4:**
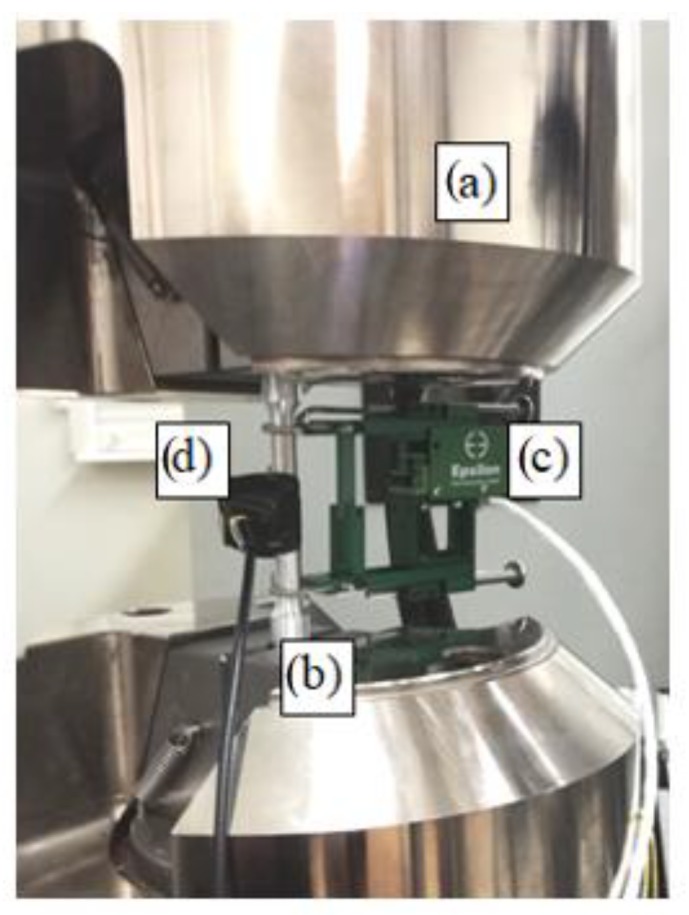
View of the specimen fixed to the tensile testing machine grips: (**a**) tensile testing machine grips, (**b**) specimen, (**c**) extensometer, (**d**) AE sensor.

The instruments consisted of: the AMSY-5 measurement system (Vallen Systeme GmbH, Icking, (Munich), Germany): M6-2 by Vallen (Icking, (Munich), Germany), with six ASIP-2 measurement cards for recording and processing the AE signal; sensors: VS150-RIC, VS160-NS, VS375-M(Vallen Systeme GmbH, Icking, (Munich), Germany ). Research AE was performed using a set consisting of single channel recorder USB AE Node, type 1283 with bandpass 20 kHz–1 MHz, preamplifier with bandpass 75 kHz–1.1 MHz, AE-Sensor VS 150M (with a frequency range of 100–450 kHz), computer with AE Win for USB Version E5.30 software (Vallen Systeme GmbH, Icking, (Munich), Germany ) for recording and analyzing AE data. Between the sensor and a surface of the specimen a coupling fluid was used. An AE Sensor was fixed to specimen by elastic tape. Parallel measurements applying the AE method were also conducted during the static tensile test. For monitoring tensile test of the chosen specimens Physical Acoustics Company (PAC) acoustic emission system was used. The software used in the AE data acquisition was AMSY–5 (Vallen Systeme GmbH, Icking, (Munich), Germany). View of the specimen fixed to the tensile testing machine grips is presented in Figure 4.

The test consisted of processing and treating acoustic signals in the source of stretching. A series of parameters were analyzed and compared, including amplitude, frequency of signals, number of computing operations, accumulated energy, RMS, duration of signals, and computing pace. These parameters referred to stress values in time. Diagrams were developed to illustrate changes in the values of selected parameters. Due to the ampleness of test results, the article only presents model diagram courses. Basing on the parameters selected for the test, the focus was on the interpretation of the number of computing operations concerning exceeded EA signal discrimination levels, the RMS mean effective electrical signal value, and the energy of AE signals. According to the authors, these parameters were used to determine the acoustic characteristics of the material under the influence of progressing degradation.

In the course of the static tensile test, the results were recorded by a universal testing machine. These results were then processed to determine the Kolmogorov-Sinai metric entropy.

In this method, the operation of the machine–sample system and the formation of measurement distortions generally stem from the non-uniformity of the deformation field and stress related to the structure of the sample material, its shape, and its fastening. Non-linear machine–sample parameters are synergistic variables, which affect one another greatly due to the feedback from the system. Damage of the sample material initiates a local process that significantly affects the dynamic characteristics of the system [24].

To illustrate the discrete distribution of likelihood, metric entropy diagrams were drawn for each of the samples as a function of time. Next, the diagrams were applied to their corresponding tensile curves.

The following results were ultimately obtained: AE method measurements and K-S metric entropy calculations. These values corresponded to the yield points of the tested material. The static tensile tests were carried out for a constant force value (force control).

The test results obtained from these three methods were used to analyze and verify the usability of these methods in determining the passage of the material structure from the elastic state to the plastic state, i.e., in determining the yield point of the material.

## 3. Results and Discussion

**Figure 5:**
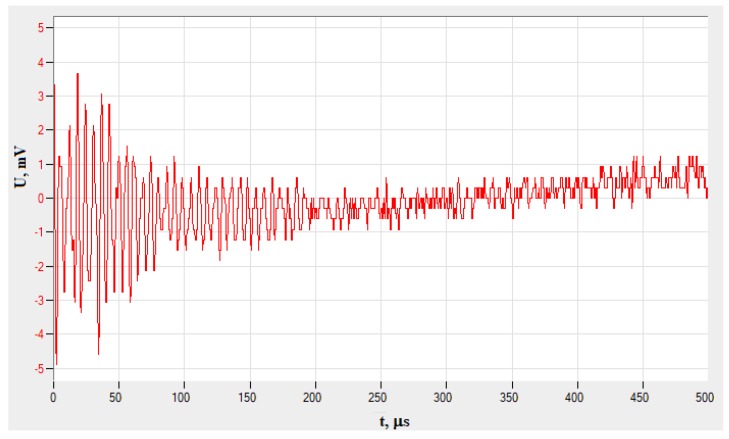
An example of an AE signal recorded during tensile test of Sample No. 3 (PAC system).

The first part of the study analyzed the results developed on the basis of acoustic emission. During the study, the AE generated by internal friction, cracking of matrix and fibers into specimen, carried out on a test stand, recorded a number of parameters, which were analyzed. These parameters were amplitude, number of events–hits, energy, RMS of the signal. The analysis of these parameters was done using AE Win for USB Version E5.30 software. For comparing the results obtained during the tests, the parameter Root–Means–Square (RMS) was chosen. A chart visualizing change in signal amplitude as a function of time, recorded during the test of Sample No. 3, is shown in Figure 5.

**Figure 6:**
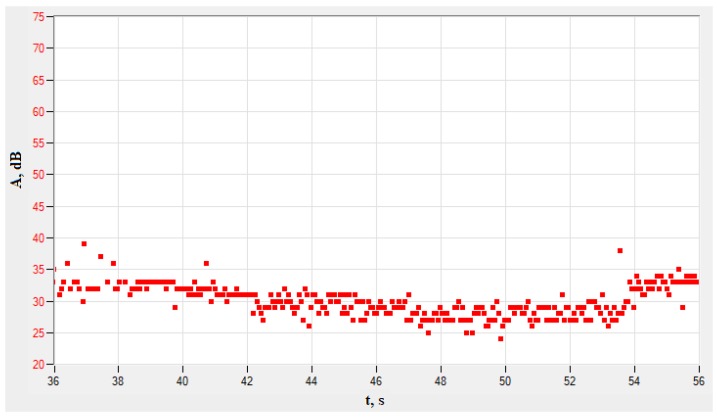
An example of changing average amplitude value as a function of time for Sample No. 3 (PAC system).

The diagram evaluates parameters as a function of time. Depending on knowledge of the analyzed materials, based on the wavelength and its amplitude, the type of damage can be characterized. The test results can be presented graphically as a change in mean value of the amplitude over time. Figure 6 presents a diagram of amplitude change as a function of time. The accumulation of points corresponds to the number of single events—signals (Figure 5). 

The amplitude values reflect the characteristics of the events that occur when loading the material. The higher the amplitude values, the greater the changes occurring in the material. Figure 6 presents the range of amplitude after exceeding the time corresponding to the yield point; hence, relatively constant values of amplitudes as a function of time are visible.

**Figure 7:**
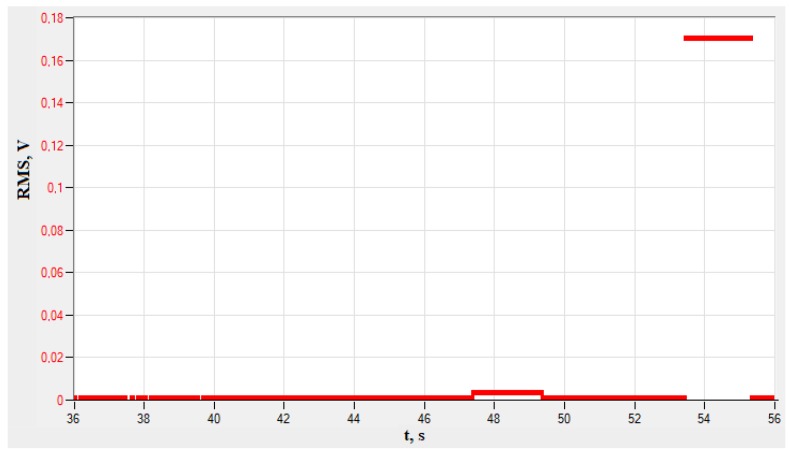
An example of changing average value of RMS as a function of time (PAC system)

Figure 7 shows the mean RMS values of the signal as a function of time. 

Figure 7 presents a graph of the root mean square of an electric signal as a function of time. The RMS is a parameter proportional to the square root of the amount of energy carried by the wave of acoustic emissions. Analyzing the obtained values of the RMS parameter, a small increase in its size to 47 s is visible at 0.014 V. This corresponds to the beginning of permanent deformations occurring in the material (yield point). Another increase is noticeable at 0.17 V, and corresponds to the material's tensile strength. In isotropic materials, it is characteristic that on the RMS (t) charts, changes occurring in the material, such as yield strength and strength, are noticeable. At the elastic limit, as well as after exceeding the yield point, there are no noticeable changes in the mileage. This means that the acoustoelectric sensor does not register the increased energy transmitted by the acoustic emission wave.

The mean values of selected characteristic values describing the signal recorded in the course of the test are presented in Table 2. Additionally, the values of the signal parameter, recorded in the course of the test, are provided. No changes are visible. A signal with such parameters is background noise from the drive of the universal testing machine and the environment. The parameters of signals recorded in characteristic moments, resulting in clear changes of the recorded values, were also presented. Signal parameters of the greatest diagnostic importance were selected. To develop metric entropy, acoustic emission, and deformation diagrams as a function of time, RMS values are expressed in mV.

An analysis of the recorded data in a specific time interval in the course of a static tensile test indicated an increase of signal amplitude from 33 dB to 70 dB. An increase of the RMS signal by 40% was observed in the same interval. Changes in parameters of the recorded signal may point to a change in material structure, i.e., the passage of the material from an elastic state to plastic state.

When the tested material reached the arbitrary yield point R_0.2_ (YP), i.e., passed from an elastic state to a plastic state, the RMS signal increased by 40% relative to the signal recorded at the beginning of the tensile test. When the tested material reached its R_m_ ultimate tensile strength (UTS), the recorded RMS signal was 170 times higher than that recorded at the beginning of the test.

The signal recorded when the material exceeded its yield point was characterized by a clear increase in the number of counts in which the discrimination threshold—number of events—was exceeded. This parameter informs about the number of events during loading. In the absence of activity, a reduced number of events is noticeable—1435, corresponding to the noise from the testing machine, as well as small deformations in the elastic range. When the yield point value is reached, the number of events increases to 2616, as well as the amplitude. It can thus be stated that irreversible changes occur in the material. When reaching strength, the number of events is smaller than that at the yield point, given that the material has already deformed plastically and tends to be damaged, with a constant increase in load; however, the energy carried by the wave of acoustic emissions is much higher, which shows the effective value of the emission signal acoustic.

**Figure 8:**
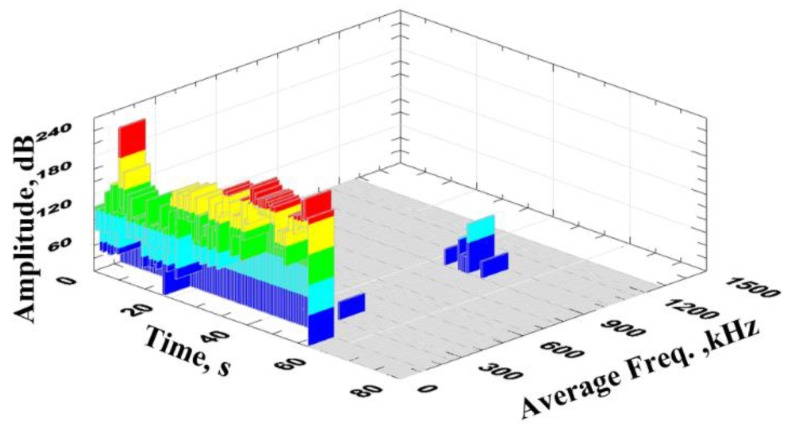
Amplitude and average frequency change as a function of time

Figure 8 presents signal amplitude and frequency change as a function of time.

**Figure 9:**
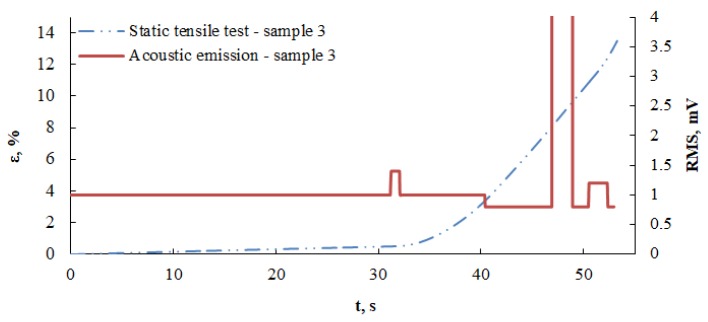
Diagram of RMS value and deformation as a function of time for Sample No. 3

Relatively high momentary changes in signal amplitude are observed after the yield point is crossed, which was not observed in the changes of mean values. An analysis of signal frequency can be used to explicitly designate the initial moment, in which the material started to plasticize. This phenomenon is accompanied by the formation of a high-frequency signal (above 1 MHz). Figure 9 presents a model diagram of deformation ε(t) and effective signal (RMS (t)) as a function of time for Sample No. 3.

The first local maximum value of the effective signal (RMS) is visible. This value was recorded in a time interval between 31.249 s and 32.053 s. A sample deformation was simultaneously recorded in the same interval. The maximum value of this signal is 1.4 mV. At 31.249 s of sample stretching, the deformation is ε = 0.496, to which the stress value refers, whereas at 32.053 s, the deformation is ε = 0.518 and corresponds to the value of the stress σ = 343.90 MPa (stress values were determined on the basis of tensile diagram σ - ε from the universal testing machine). According to AE characteristics recorded in deformation conditions, the first RMS maximum after the material has surpassed the threshold for permanent deformations corresponds to the yield point, and the second maximum occurs for large deformations [29]. Analyzing the deformation diagram as a function of time, one can observe a characteristic property—from the first RMS maximum, elastic deformation takes place in the material (which is not visible in the diagram), after surpassing the first RMS maximum. As the tensile process progresses, the deformation clearly increases. The first RMS maximum is the moment when the first permanent deformations appear in the material, signifying loss of elasticity. This corresponds to the end of yield point. The second RMS maximum occurs after significant deformation of the sample and is related to the dynamic development of destruction in the material.

The second part of the study analyzed the results developed on the basis of the K-S metric entropy method.

Metric entropy calculations (the entropy of measurement data) are performed based on the results of the static tensile test, i.e., deformation as a function of time. The procedure for calculating the K-S entropy consists of dividing the data into a correct number of sets of digits. For instance, Table 3 includes one full 50-digit interval obtained from a collection of absolute elongation values in the course of a test of sample. The numerical ranges adopted include subsequent displacement values obtained with the Epsilon 3542 extensometer. The displacement values obtained at the measuring points from No. 101 to 150 were used for further analysis. The lowest recorded value in this number range is 0.001951947 mm, and the highest 0.002047 mm. The interval was divided into four one-sided closed sub-compartments (from 1 and = to N = 4), marked in Table 3 as columns: I, II, III, IV. Then, the probability ip was calculated so that the element of the accepted 50-digit interval is in the appropriate sub-compartment (Table 3). The probability calculated for each of the ip subbands was finally used to calculate the S metric entropy for the selected 50-digit interval, according to the following relationship [28,29,33]:

(3) $$S=-\sum_{i=1}^{N}p_{i}\times \ln p_{i}=-(-0.3502-0.3425-0.3502-0.3425)=1.3855$$

The value of the calculated entropy S was assigned to the middle point of the assumed range, i.e., for measuring point No. 125. In order to calculate the metric entropy S for the whole set of recorded values of the sample elongation, calculations of the abovementioned method for all 50 numerical intervals of this set were made. The number of recorded measurement points for the analyzed sample is 3300. Therefore, the metric entropy should be calculated for 3300 − 50 = 3250 intervals. The deformation value corresponding to the measuring point where the minimum metric entropy occurs corresponds to the yield point of the material.Using the results of the static tensile test carried out on the EN AW 7020 alloy specimen, the K-S metric entropy method was applied in determining the yield point of the tested material. 

**Figure 10:**
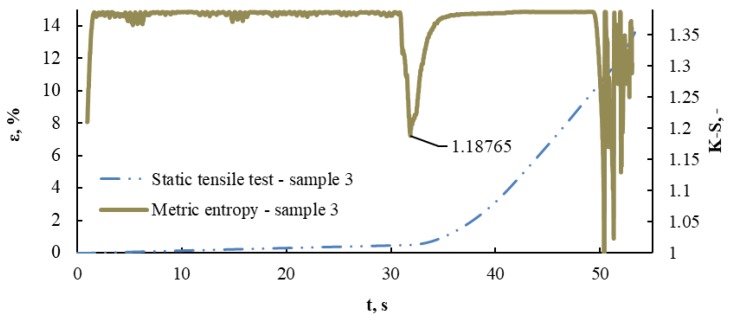
Diagram of metric entropy K-S and deformation ε as a function of time for Sample No. 3.

Figure 10 presents a diagram of K-S metric entropy and deformation as a function of time for Sample No. 3

A local metric entropy minimum corresponding to 1.18765 has been identified. This minimum occurred in the 1597th measurement point corresponding to 31.842 s, for which the deformation value is ε = 0.508, and the stress value is σ = 340 MPa. K-S metric entropy is a procedure that uses measurement data, treating the yield point as a “critical point” that separates the state of elasticity from the plastic state in the process of deformation of the construction material. In this method, a decrease in the metric entropy value at the yield point is expected, caused by the non-linearity associated with the change in the data structure near this transition from the elastic to plastic state, increased by fluctuations associated with the occurrence of deterministic chaos.

The recorded decrease in entropy is closely related to the energy dissipation in the sample being tested, and therefore to the noticeable change in the dynamics of data recorded by the universal testing machine. A change in data dynamics is the response of a material to the load it is exposed to, and the structural changes that occur in it. Hence, the entropy value of 1,187,65 indicates a transition from elastic to plastic in the tested material.

**Figure 11:**
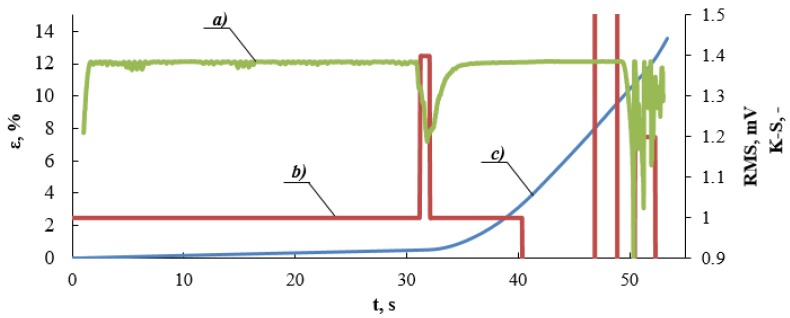
Diagram of (**a**) metric entropy K-S, (**b**) value of effective electrical signal RMS, (**c**) deformation ε as a function of time.

Figure 11 presents a diagram of K-S metric entropy, value of the effective signal RMS, and deformation ε as a function of time. 

**Figure 12:**
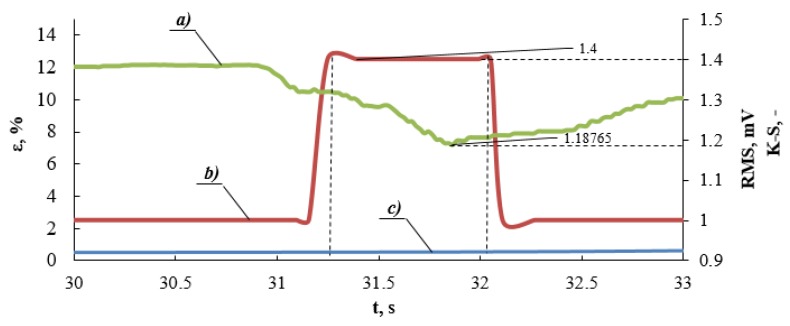
Diagram of (**a**) metric entropy K-S, (**b**) value of effective electrical signal RMS, (**c**) deformation ε as a function of time (a part of the tensile diagram—Figure 11).

This figure presents the course of characteristics in time. The value of the first RMS maximum, which corresponds to the minimum value of metric entropy, is presented in the figure. These values are assigned to deformation determined on the basis of the static tensile test (Figure 2, Figure 9 and Figure 10). Figure 12 presents a zoom in on selected fragments of diagrams presented in Figure 11.

**Figure 13:**
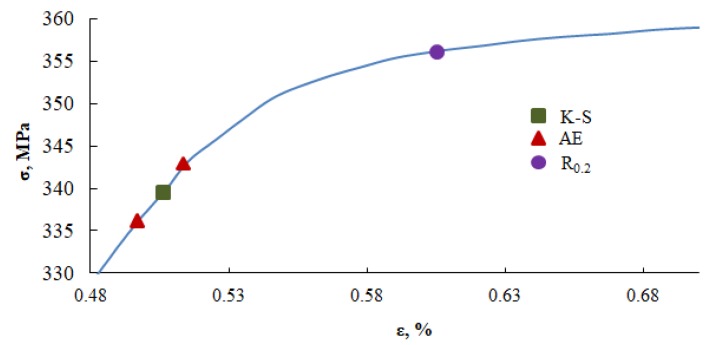
A fragment of the static tensile diagram for Sample No. 3 (Figure 2), with marked values of the yield point determined using the K-S metric entropy method, K – S ; the first local RMS maximum; the end local RMS maximum of acoustic emission (AE), with a marked value of R_0.2_, determined in the static tensile test.

The maximum RMS and minimum values of metric entropy are marked for the analyzed Sample N. 3. The value of the RMS parameter fits into a specific time interval, whereas the value of metric entropy corresponds to a specific point. Figure 13 presents a diagram obtained in the static tensile test carried out for Sample No. 3, where the values of the yield point was obtained using three methods: the yield strength determined using the K-S metric entropy method −340 MPa, the AE method for the first local RMS maximum −336 MPa for the end of the first RMS maximum −344 MPa, and on the basis of the tensile diagram R_0.2_ = 356 MPa. The range of maximum RMS (mV) values, as well as the minimum K-S entropy values, determines the transition from the elastic phase to the plastic phase in the tested material. Figure 13 presents a summary of the results obtained from the tests carried out graphically, while Table 4 presents a summary.

Figure 13 presents the results obtained from the testing machine by the tangential method, by the acoustic emission method, as well as from K-S metric entropy. On their basis, it can be determined that yield strength, determined conventionally, slightly differs from the values obtained by the acoustic emission method (approx. 4%), and thus the K-S metric entropy. Due to the fact that acoustic emission is based on elastic waves, it is not possible to present it as one point, but as a range of values. For all tested samples, the values obtained by the K-S metric entropy method are in this range. Table 4 summarizes the results obtained.

Table 4 lists the results obtained for the tested samples.

The results of strength tests presented in Table 4 indicate that the yield point values obtained for individual samples using the K-S metric entropy method are similar, and that the yield point corresponds to a strictly defined deformation value. With the AE method, due to its specific, wavy character, the analysis produces a deformation interval, which is then used to determine the yield point interval. 

Both methods, i.e., the acoustic emission method and the K-S metric entropy method, can be used to successfully determine changes occurring in the structure of a material, which passes from the elastic phase to the plastic phase. The methods presented provide an accurate determination of the beginning of permanent deformation in the material after it has surpassed its yield point.

## 4. Conclusions

The notion of yield point determination, that is the determination of the point where a material passes from the elastic phase to the plastic phase, is pertinent to all materials, characterized by the lack of clear yield strength. 

The acoustic emission method is a known and commonly used method for diagnosing technical objects as well as for determining the mechanical properties of materials in which elastic waves are created and propagated. In the case of samples subjected to tension, the results related to the transition of the material from elastic to plastic state are recorded. However, the acoustic emission method requires specialized, very expensive equipment, which is presented in the Materials and Methods chapter. However, the K-S metric entropy method allows similar results to be obtained, using a simple mathematical apparatus. Modern testing machines allow obtaining data that can be processed accurately. The application of the K-S metric entropy results in enables satisfactory results, allowing the engineer to use them for the design of structures.

The study has proven that:

1. The yield point—there was a clear decrease in the metric entropy value caused by nonlinearities associated with the change in data structure in the area of transition from the elastic phase to the plastic phase
2. The application of the Kolmogorov-Sinai metric entropy allows the determination of material constants of construction materials as a result of analyzing the dynamics of the internal structure of measurement data
3. Both the acoustic emission method and K-S metric entropy allow the yield point to be determined with high accuracy, while the K-S metric entropy method does not require specialized equipment, only preparation of a calculation program
4. By using the acoustic emission method, the range of values for the yield point can be determined, while K-S metric entropy allows for accurate determination of the transition from the elastic phase to the plastic phase within the AE range.
5. Determination of material constants based on metric entropy in a static tensile test enables detailed examination of deformations of composite materials of complex structure.

## Figures and Tables

**Table 1:** Chemical composition of the EN AW 7020 alloy sheet metal of g = 12 mm, certificate no.: 17050-1, cast no.: 1-12-9007.

Fe	Si	Ti	Zr	Zn	Mg	Mn	Cu	Cr	Other	Al
%										
min 0.40	max 0.35	0.08–0.25	0.08–0.20	4–5	1–1.4	0.05–0.50	max 0.20	0.10–0.35	max 0.15	residual

**Table 2:** Values of chosen parameters recorded in characteristic moments during the test for Sample No. 3.

Condition of the Specimen	Counts -	Amplitude (dB)	RMS (mV)
No activities	1435	33	1.0
Yield point (YP)	2616	71	1.4
Ultimate tensile strength (UTS)	2189	34	170.2

**Table 3:** Procedure of computing the K-S metric entropy from simulated data.

Number of Sub-Compartments	I	II	III	IV
min	0.001947957	0.001973781	0.001998242	0.002025
max	0.001949942	0.001975782	0.002000261	0.002027
1	0.001951947	0.001978075	0.00200224	0.002029
2	0.001954014	0.001980329	0.002004362	0.002031
3	0.001956054	0.00198233	0.002006428	0.002033
4	0.001958079	0.001984359	0.002008444	0.002035
5	0.001959842	0.00198639	0.002010482	0.002037
6	0.001961893	0.001988331	0.002012511	0.002039
7	0.001963651	0.001990307	0.002014748	0.002041
8	0.001965724	0.001992233	0.002016782	0.002043
9	0.001967753	0.001994258	0.002018736	0.002045
10	0.001969754	0.001996199	0.002020994	0.002047
11	0.001971706	-	0.002023023	-
*p_i_*	0.26	0.24	0.26	0.24
ln*p_i_*	−1.347073648	−1.427116356	−1.347073648	−1.42712
*p_i_* ln*p_i_*	−0.350239148	−0.342507925	−0.350239148	−0.34251
*S*	1.385494148			

**Table 4:** A list of yield point values obtained with the K-S metric entropy method, effective RMS signal, the AE method and based on the static tensile test diagram for samples made of EN AW 7020 aluminum alloy.

Sample No.	Yield Point Obtained with the K-S Metric Entropy Method MPa	Yield Point Obtained with the AE Method for the Beginning of the First RMS Maximum, MPa	Yield Point Obtained with the AE Method for the End of the First RMS Maximum, MPa	R_0,2_ Obtained with the Static Tensile Test Method, MPa
1	338	298	340	353
2	339	326	342	354
3	340	336	344	356
Mean value	339	320	342	354

## References

[1] Krampikowska A., Pała R., Dzioba I., Swit G. (2019). The Use of the Acoustic Emission Method to Identify Crack Growth in 40CrMo Steel. Materials.

[2] Logoń D. (2019). Identification of the Destruction Process in Quasi Brittle Concrete with Dispersed Fibers Based on Acoustic Emission and Sound Spectrum. Materials.

[3] Tai J., He T., Qiang P., Zhang D., Wang X. (2019). A Fast Beamforming Method to Localize an Acoustic Emission Source under Unknown Wave Speed. Materials.

[4] Máthis K., Chmelík F., Janecek M., Hadzima B., Trojanová Z., Lukáč P. (2006). Investigating deformation processes in AM60 magnesium alloy using the acoustic emission technique. Acta Mater..

[5] Vinogradov A., Orlov D., Danyuk A., Estrin Y. (2013). Effect of grain size on the mechanisms of plastic deformation in wrought Mg–Zn–Zr alloy revealed by acoustic emission measurements. Acta Mater..

[6] Grosse C.U., Ohtsu M. (2008). Acoustic Emission Testing.

[7] Renachowski Z. (2012). Emisja akustyczna w diagnostyce obiektów technicznych. Drogi Mosty.

[8] Dudzik K., Charchalis A. (2015). Wykorzystanie emisji akustycznej do diagnozowania wtryskiwacza. Logistyka.

[9] Miller R. (1994). Acoustic emission testing of storage tanks: Tappi Journal, Vol. 73, No. 12, pp. 105–109 (Dec. 1990). NDT E Int..

[10] Gołaski L. (1994). Emisja akustyczna w materiałach złożonych. Emisja Akustyczna.

[11] Ziegler B., Miszczak A. (2017). Acoustic emission as a friction force indicator after test stands. J. KONES.

[12] Ziegler B. (2009). Praca Doktorska: Zastosowanie Emisji Akustycznej do Identyfikacji Warunków Smarowania łożYsk.

[13] Sutowski P., Plichta S. (2006). An investigation of the grinding wheel wear with the use of root-mean-square value of acoustic emission. Arch. Civ. Mech. Eng..

[14] Ziegler B. (2007). Contribution of acoustic emission into optimal bearing lubrication. J. KONES.

[15] Goszczyńska B., Swit G., Trąmpczyński W., Krampikowska A., Tworzewska J., Tworzewski P. (2012). Experimental validation of concrete crack identification and location with acoustic emission method. Arch. Civ. Mech. Eng..

[16] Al-Jumaili S.K., Pearson M., Holford K., Eaton M., Pullin R. (2016). Acoustic emission source location in complex structures using full automatic delta T mapping technique. Mech. Syst. Signal Process..

[17] Chobola Z., Korenska M., Pazdera L., Smutny J., Šikula J., Weber Z. (2000). Non-destructive testing method of acoustic emission with generated load. Phys. Prop. Mater..

[18] Lugo M., Jordon J.B., Horstemeyer M.F., Tschopp M., Harris J., Gokhale A. (2011). Quantification of damage evolution in a 7075 aluminum alloy using an acoustic emission technique. Mater. Sci. Eng. A.

[19] Chang H., Han E.-H., Wang J., Ke W. (2009). Acoustic emission study of fatigue crack closure of physical short and long cracks for aluminum alloy LY12CZ. Int. J. Fatigue.

[20] McCrory J., Al-Jumaili S.K., Crivelli D., Pearson M., Eaton M., Featherston C.A., Guagliano M., Holford K., Pullin R. (2015). Damage classification in carbon fibre composites using acoustic emission: A comparison of three techniques. Compos. Part B Eng..

[21] Karczewski R., Płowiec J., Spychalski W., Zagórski A. (2011). Charakterystyki sygnałów akustycznych podczas obciążania wybranych stali konstrukcyjnych wykorzystywanych do budowy urządzeń ciśnieniowych. Przegląd Spaw..

[22] Świt G., Trąmpczyński W., Krampikowska A. (2015). Badania stalowych podziemnych zbiorników bunkrowych z wykorzystaniem metody emisji akustycznej, Diagnostyka w ocenie bezpieczeństwa konstrukcji, XXVII Konferencja Naukowo-Techniczna Awarie budowlane. Szczecin.

[23] Moliński W., Raczkowski J. (1998). Aktywność emisji akustycznej podczas mechanicznie wymuszonego pękania drewna w poprzek włókien. Folia For. Pol. B.

[24] Amigó J.M., Kennel M.B., Kocarev L. (2005). The permutation entropy rate equals the metric entropy rate for ergodic information sources and ergodic dynamical systems. Phys. D Nonlinear Phenom..

[25] Garbacz G. (2008). Rozprawa Doktorska: Przetwarzanie Danych Doświadczalnych z Uwzględnieniem ich Chaotycznego Charakteru.

[26] Racine J., Maasoumi E. (2007). A versatile and robust metric entropy test of time-reversibility, and other hypotheses. J. Econ..

[27] Kawan C. (2014). Metric Entropy of Nonautonomous Dynamical Systems. Nonautonomous Dyn. Syst..

[28] Kolmogorov A.N. (1959). Entropy per unit Time as a Metric Invariant of Automorphism. Dokl. Russ. Acad. Sci..

[29] Sinai Y.G., Sinai Y.G. (2010). On the Notion of Entropy of a Dynamical System. Zeichenhorizonte.

[30] Tempczyk M. (2002). Teoria Chaosu dla Odważnych.

[31] Dietrich L., Garbacz G. (2004). Uwzględnienie Chaosu w Analizie Danych Doświadczalnych, XXI Sympozjum Mechaniki Eksperymentalnej Ciała Stałego, Jachranka.

[32] Garbacz G., Kyzioł L. (2017). Application of metric entropy for results interpretation of composite materials mechanical tests. Adv. Mater. Sci..

[33] Kyzioł L., Hajdukiewicz G. (2019). Application of the Kolmogorov-Sinai Entropy in Determining the Yield Point, as Exemplified by the EN AW-7020 Alloy. J. Konbin.

[34] Pilecki S. (1994). Emisja Akustyczna: Źródła, Metody, Zastosowania.

[35] Panasiuk K. (2019). The use of acoustic emission signal (AE) in mechanical tests. Przegląd Elektrotechniczny.

[36] PN-EN ISO 6892-1:2016-09-Metals—Tensile Test—Part 1: Test Method at Room Temperature.

